# Stamp Technique: An Explorative SEM Analysis

**DOI:** 10.3390/dj11030077

**Published:** 2023-03-08

**Authors:** Francesca Zotti, Stefano Vincenzi, Alessandro Zangani, Paolo Bernardi, Andrea Sbarbati

**Affiliations:** 1 Department of Surgical Sciences, Pediatrics and Gynecology, University of Verona, P.le L.A.Scuro, 10, 37134 Verona, Italy; 2 Private Practice, 37134 Verona, Italy; 3 Department of Neuroscience, Biomedicine and Movement, Human Anatomy and Histology Section, University of Verona, Strada Le Grazie, 8, 37134 Verona, Italy

**Keywords:** restorative dentistry, stamp technique, dental materials

## Abstract

Background: Achieving correct tooth anatomy and saving time at the dental chair are some of the goals of modern restorative dentistry. Stamp technique has gained acceptance in clinical practice. The aim of this study was to evaluate the effectiveness of this technique in terms of microleakage, voids, overhangs and marginal adaptation of Class I restorations, and to analyse the operative times in comparison with traditional restorative procedures. Methods: Twenty extracted teeth were divided into 2 groups. Ten teeth in the study group (SG) were Class I prepared and restored using stamp technique, and ten teeth in the control group (CG) were Class I restored traditionally. SEM analysis was performed to evaluate voids, microleakage, overhangs, and marginal adaptation, and operative times were recorded. A statistical analysis was performed. Results: There were no significant differences in microleakage, marginal adaptation and filling defects between the two groups, however, the stamp technique seems to facilitate the formation of large overflowing margins that require a careful finishing phase. Conclusions: Stamp technique does not seem to have any critical aspects in terms of restoration durability and it can be performed in a short time.

## 1. Introduction

One of the goals of a dental restoration is to achieve the correct occlusal anatomy. This goal is not so easy to achieve with the traditional incremental technique, as training and technical skills can be an obstacle to this [1,2]. However, occlusal stability is of fundamental importance for tooth replacement, for a good neuromuscular balance and comfort during mastication [3]. To avoid these complications, a restorative technique known as stamp technique was proposed with the aim of making the design of the occlusal anatomy faster and more reproducible and minimising anatomical error [4]. The stamp technique consists of creating an occlusal matrix before removing the carious tissue in order to imprint the occlusal anatomy of the posterior teeth. This matrix is pressed onto the final composite increment before light curing. This technique is suitable for cases where caries is obvious on clinical examination.

The literature highlights that this technique is useful in reproducing the original occlusal anatomy and occlusion. It requires minimal finishing and polishing and allows for minimal voids and a desirable polymerised occlusal surface [1,5,6]. Moreover, it seems that it does not require much experience to perform effective restorations in a short time [4]. Certainly, the stamping technique is a protocol with a very narrow indication and is therefore aimed at carefully selected clinical conditions, such as posterior carious or erosive lesions with undamaged occlusal and cusp surfaces.

In addition, the literature suggests further use of the punch technique also for interproximal carious lesions where the occlusal and interproximal walls and marginal ridge are intact [1]. Nevertheless, the use of stamp technique is reported in the restoration of anterior teeth, provided that the original shape of the teeth is preserved [1]. All the proposed applications of the technique are considered useful for restoring proper anatomy and saving operative time.

Although no clear protocol can be found in the literature, case reports satisfactorily demonstrate the clinical steps for performing restorations with the stamp technique. Essentially, there are two distinct phases between the traditional incremental technique and the stamp technique. The first phase consists of making the occlusal matrix (stamp) before cavity preparation and before [7] or after [1] isolating the surgical field. It requires the following steps:

- Cleaning and drying the occlusal surface with a brush and airflow to reduce the interference of plaque, food debris and saliva [7]
- The application of release agents, such as petroleum jelly [4,7], to make the stamp easy to remove after light curing and to make the shape of the stamp more regular, as the release agents penetrate into the deep pits and fissure, which are functionally useless spaces [1]
- The actual fabrication of the stamp consists of applying a material with good plasticity evenly to the occlusal surface and fitting it into the pits and fissures and over the cusps to create an impression of the surface that can be used in the final phase of the restoration. The most commonly used materials for making the impression are flowable composite [1,4], liquid dental dam [8], or acrylic resin [7]. They must allow light to pass through during the light-curing of the final layer of the restoration. Before the polymerisation of the material is complete, a microbrush is used to make a stem for the final phase of the restoration [1]. The market offers special devices for this purpose. They have a handle and a cartridge made of polyethylene, which, heated, is pressed against the tooth to create the impression [9]. At the end, the light-cured stamp is removed, taking care not to damage it.

The phase of using the stamp occurs in the final phase of the restoration to imprint the occlusal layer of composite. The clinical steps are as follows:

- The application of the final layer of composite, 1–2 mm thick, to the occlusal surface [1,7,9]
- The application of a release agent that prevents adhesion between composite resin and stamp; the most commonly used material is Teflon [1]. However, the literature reports also the use of transparent film [4] or petroleum jelly [7]
- The matrix is pressed against the surface in the correct position to allow the stamp to adapt to the not yet cured composite [6]
- The light-curing of the last increment of composite that passes through the stamp ensures a perfect adaptation of the occlusal surface to the matrix and the original occlusal morphology. After removing the punch, another light-curing step is recommended [7]
- Finishing and polishing of the restored surface, especially of the tooth-composite portion [1,9]

The aims of this in vitro study were:

- To analyse the performance of the stamp technique compared to the traditional increment technique in terms of microleakage, marginal adaptation, overhangs and voids of Class I restorations using the scanning electron microscope (SEM);
- To evaluate the impact of the stamp technique on operative times in the fabrication of Class I restorations compared to the traditional increment technique.

## 2. Materials and Methods

Eighteen human molars and two premolars, both maxillary and mandibular, were extracted undamaged for periodontal reasons before February 2020. Inclusion criteria for the in vitro study were no carious lesions or other changes affecting occlusal morphology and integrity.

After tooth extraction, teeth were polished and disinfected in a 0.2 chloridexidine solution. To avoid dehydration, they were stored in physiological solution [10].

The teeth were divided equally into two groups: 10 of the sample group (SG), 9 molars and 1 premolar to be filled by stamp technique, and 10 of the control group (CG), 9 molars and 1 premolar to be filled with the traditional increment technique.

The SG elements were cleaned with a brush and water to create the occlusal stamp. A layer of Vaseline was applied to the occlusal surface as a separating agent and an increment of flowable composite—Estelite Flow Quick (Tokuyama Dental Italy, S.r.l., Sandrigo, VI, Italy)—was placed on the surface and a tip of a dental probe was used as a handle, then the material was light-cured according to the manufacturer’s instructions.

For all extracted teeth, a Class I black cavity prepared for composite with a depth of 4 mm and a width guaranteeing at least 2 mm residual wall width was created with a conical diamond bur for the enamel and a round carbide bur for the dentin. Occlusally unsupported enamel prisms were removed. Adhesive procedures were performed with Tokuyama Etching Gel HV (Tokuyama Dental Italy, S.r.l., Sandrigo, VI, Italy) and Tokuyama EE Bond (Tokuyama Dental Italy, S.r.l., Sandrigo, VI, Italy). The restorations were fabricated with multiple increments of Omnichroma Universal Composite (Tokuyama Dental Italy, S.r.l., Sandrigo, VI, Italy). The elements of CG were completely restored with the increment technique (oblique layering technique) [11], while those of SG were filled up to the last 2 mm of the cavity. The last 2 mm were filled with non-light-cured composite and covered with a Teflon layer against which the occlusal punch was pressed to perform the occlusal punch technique with a correct occlusal anatomy.

All restorations (SG and CG) were then finished and polished with low grit and silicone drills.

The same operator performed the cavities preparations and all the restorations, both with incremental technique and stamp technique.

All recovered samples were therefore thermocycled using the standard ISO TR 11450 (2015): 500 cycles of 30″ [12] each in water with temperature variations between 5 ± 2 °C and 55 ± 2 °C to estimate nearly two months of usage in the oral environment [13]. The time was calculated with a digital chronometer and completed in 4 h and 10 min.

For the 30″ cycle at 55 ± 2 °C, an immersion thermostat (Julabo MP-5 Heating Circulator, JULABO GmbH, Seelbach, Germany) was used for temperature control, while the 30″ cycles at 5 ± 2 °C were performed in an ice bath with constant temperature recording.

All restored and thermocycled teeth were then sectioned in the vestibular-lingual direction with a conical diamond bur under constant water irrigation. The roots of teeth were completely retained into clear acrylic resin during cutting to avoid cracking. No dyes were used.

### 2.1. SEM Analysis

The cut tooth elements were then washed with distilled water and alcohol (70% solution) and allowed to dry. They were then placed on an aluminium surface and metallised with a suitable metallising instrument (Balzers MED 010, Blazers, CAE, Austin, TX, USA) and observed from SEM (FEI/Philips XL30 ESEM).

Three scanning electron images were taken for each sectioned tooth as follows:

- One 40× image to assess the tooth-restoration interface and possible voids in the composite for full restorations;
- Two 250× images (right and left) of the coronal part of the teeth to assess the occlusal surface, marginal adaptation and overhangs.

All measurements of the defects visible in the images were made with the open source software ImageJ (National Institutes of Health, Bethesda, MD, USA).

### 2.2. Evaluation Criteria

#### 2.2.1. Microleakage

**Figure 1:**
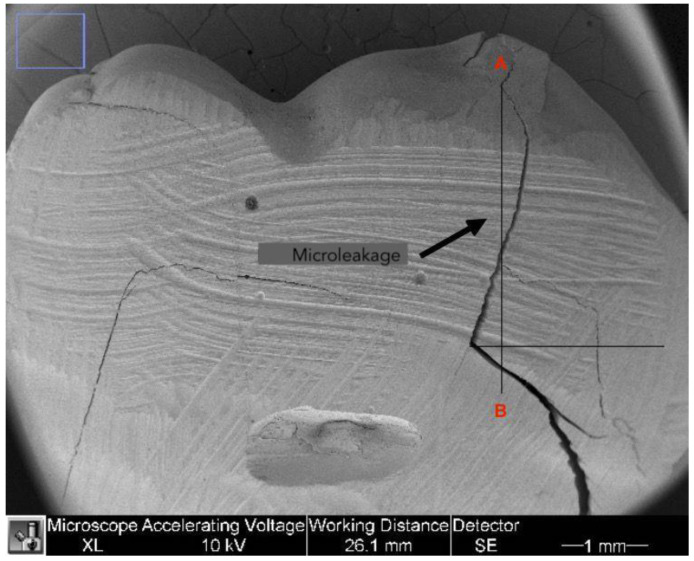
The distance between point A and point B on the 40× image.

The microleakage was assessed during analysis of the tooth-restoration interface on 40× images. The gap was considered remarkable if there was a cavity between the tooth and the composite. Different landmarks were marked to calculate the microleakage values. The most coronal point of the tooth-restoration interface was marked (point A) and a line was drawn in the apical direction. A second line was designed perpendicularly starting from the point where cavities between composite and tooth were no longer visible. The intersection between the two lines was identified as point B. The distance between point A and B was measured using ImageJ software and expressed in mm as shown in Figure 1.

These measurements were taken for both sides of the restoration (right and left) and the highest value was considered as microleakage. Given the 4 mm depth of all cavities, the author used the following formula to calculate the actual microleakage [14]:

%MicroLeak = (AB/4 mm) × 100

#### 2.2.2. Marginal Adaptation

**Figure 2:**
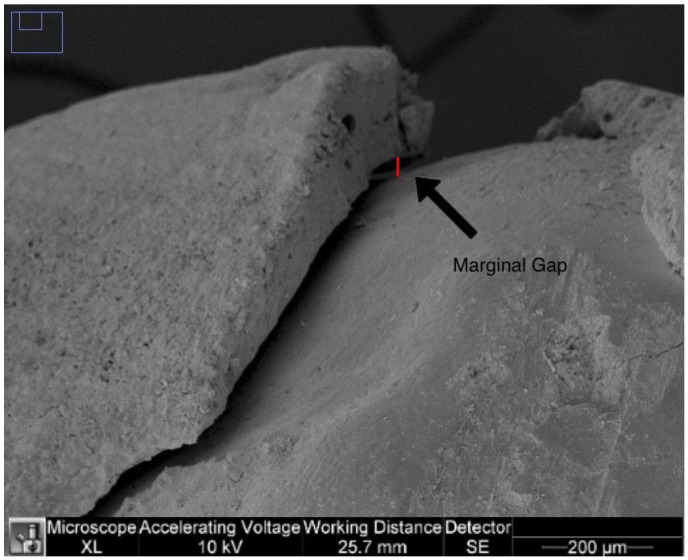
Marginal gap on a 250× image.

The marginal adaptation was calculated on 250× images of the right and left sides of the image. For each image, the largest distance between the margins of the tooth restoration (resulted by sequential measurements of the distances in µm along the visible gap) was measured in µm and the right and left marginal gaps were determined for each specimen as in Figure 2.

In addition, the highest value in µm for each tooth was used for analysis.

#### 2.2.3. Overhangs

**Figure 3:**
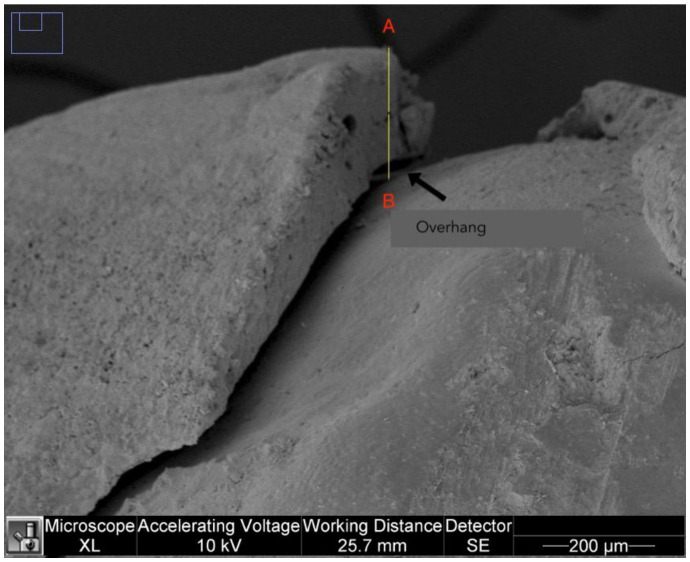
Overhang on a 250× image.

The analysis of the overhangs was carried out on 250× images of the right and left sides. A landmark was set as point A on the most overhanging margin of the restoration, from which a straight line was drawn to the tooth surface (point B). The segment AB was measured for each side and the highest value in µm for each tooth was considered for analysis (Figure 3).

#### 2.2.4. Voids

**Figure 4:**
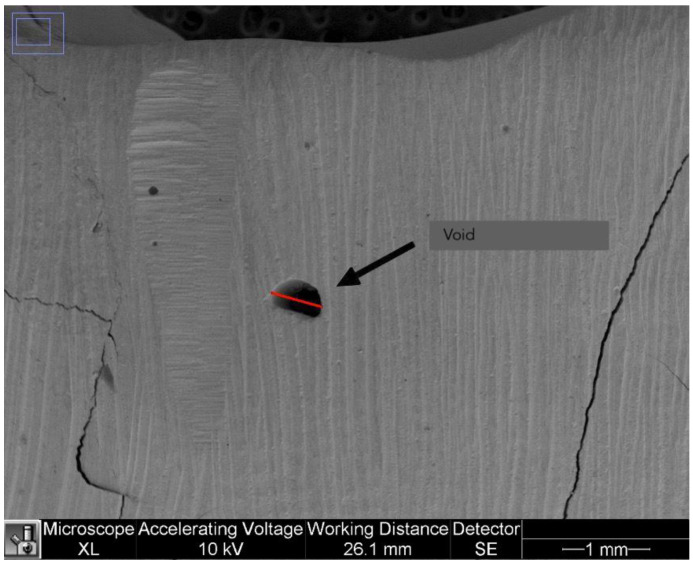
A void on a 40× image.

The presence of voids in the restorations was assessed as present/absent using the 40× images. In addition, the largest diameter of air bubbles that may have been present in the last coronal 2 mm of the resin was measured in mm. Only air bubbles wider than 2 mm were considered in the analysis. Voids were calculated in mm as shown in Figure 4.

#### 2.2.5. Operative Times

For each group, operative times were recorded in seconds by an external operator using a digital chronometer as follows:

- SG: (a) preparation of occlusal stamp (brushing of occlusal surface, application of petroleum jelly and flowable composite, light curing); (b) restorative procedures (adhesive procedures and filling with increments of composite and light curing, stamp technique).
- CG: (a) restorative procedures (adhesive procedures and filling with composite increments and light-curing).
- SG and CG: finishing and polishing. These phases were considered complete by the operator based on the clinical judgment, as in routinely practice occurs.
- The times collected were recorded in a database and divided into two categories: Restorative time and Finishing time.

### 2.3. Statistical Analysis

The data were statistically analysed as follows:

- Microleakage data were tested for normality using the Shapiro-Wilk test. The U Mann-Whitney test was then performed to assess the difference in microleakage between the two groups;
- The data of the marginal adaptation were tested for normality using the Shapiro-Wilk test and then the U Mann-Whitney test was performed to assess the difference between the two groups;
- The overhang data were tested for normality using the Shapiro-Wilk test and Levene’s test was performed to assess the homogeneity of variance. The *t*-test was used to compare the marginal overhangs between the two groups.
- The data from the voids wider than 2 mm were tested for normality using the Shapiro-Wilk test and then the U Mann-Whitney test was performed to assess the differences between the two groups;
- Operative times (restorative time and finishing time) in seconds were tested for normality using the Shapiro-Wilk test and Levene’s test was performed to assess homogeneity of variance. The *t*-test was used to compare the operative times between the two groups.

Level of significance were set at 0.05. Statistical analysis was run using Statistical Package for Social Sciences 25.0 Version (SPSS Inc., Chicago, IL, USA).

## 3. Results

Twenty Class I restorations were analysed in the present study. The restorations assigned to SG (1 premolar and 9 molars) and those assigned to CG (1 premolar and 9 molars) were tested for microleakage, marginal adaptation, presence of overhangs and voids. Operative times were tested for each filling technique.

### 3.1. Microleakage

The following tables show the microleakage data between the two groups (Table 1 and Table 2).

The U Mann-Whitney test showed no statistically significant differences between the groups (*p* = 0.288).

**Figure 5:**
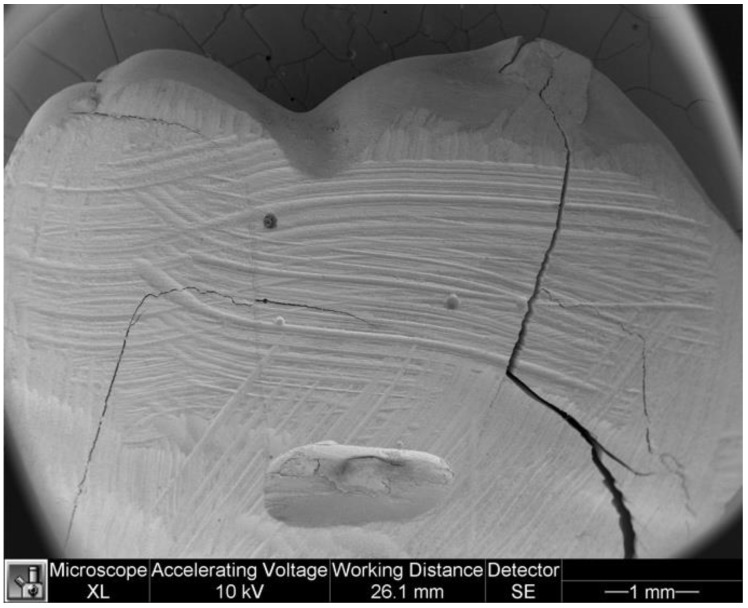
Microleakage in a specimen of study group.

**Figure 6:**
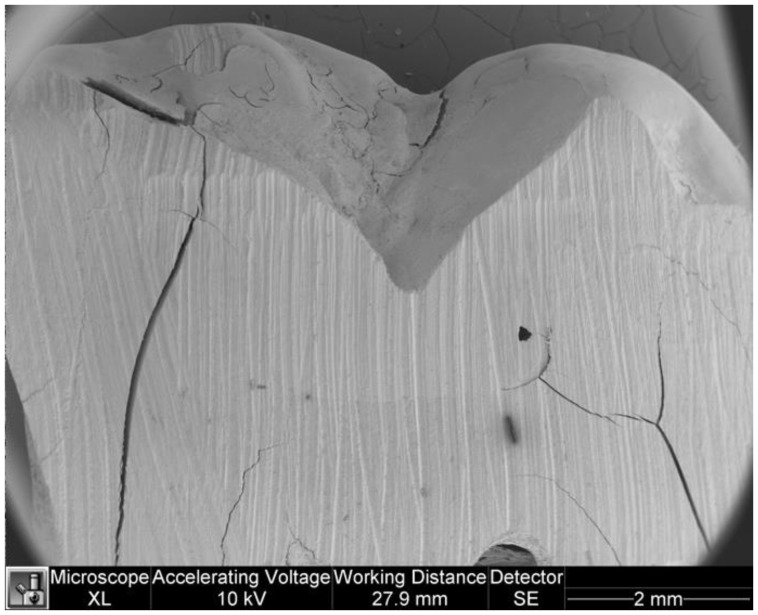
Microleakage in a specimen of control group.

Figure 5 and Figure 6 show microleakage in specimens.

### 3.2. Marginal Adaptation

**Figure 7:**
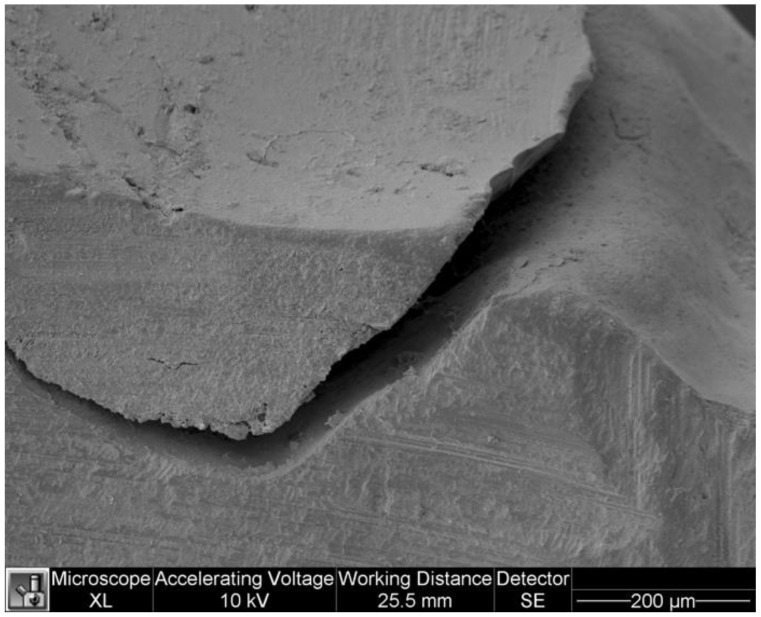
Marginal adaptation in a specimen of the study group.

**Figure 8:**
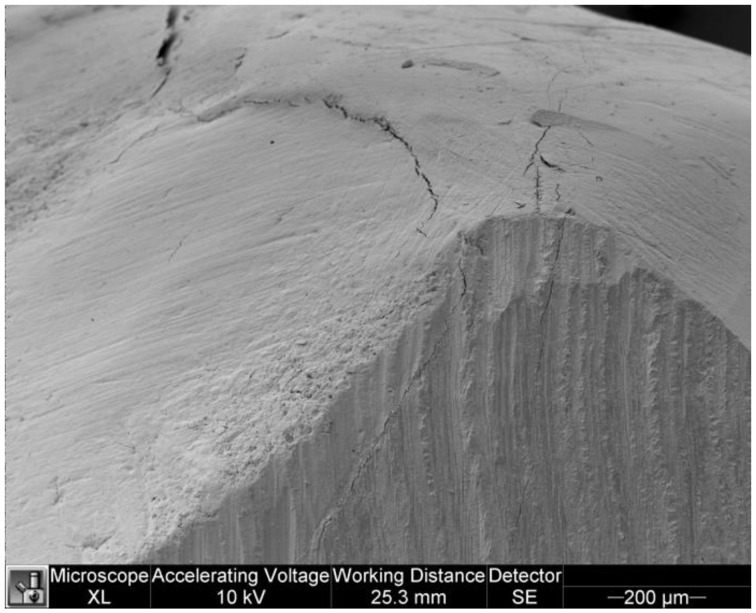
Marginal adaptation in a specimen of the control group.

The following tables show the marginal adaptation data between the two groups (Table 3 and Table 4). The U Mann-Whitney test showed no statistically significant differences between groups (*p* = 0.821). Figure 7 and Figure 8 show marginal adaptation in specimens.

### 3.3. Overhangs

**Figure 9:**
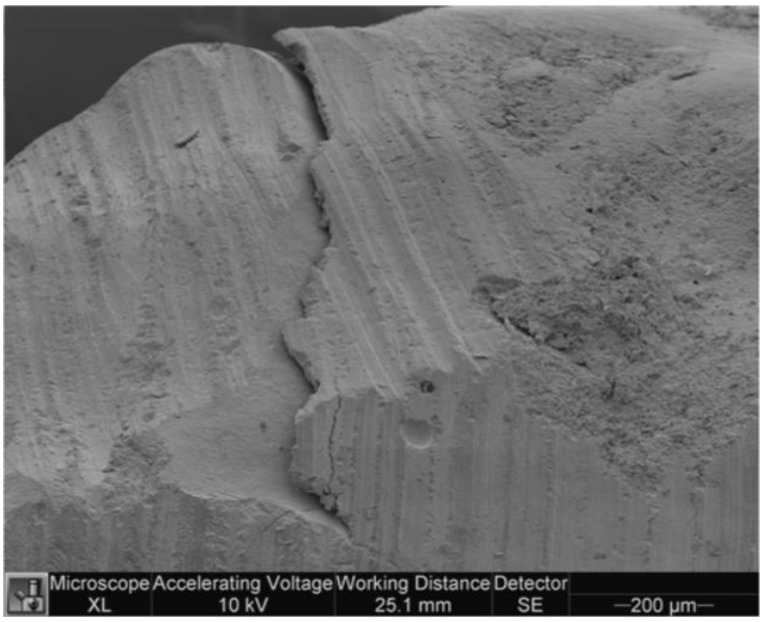
Overhang in a specimen of the study group.

**Figure 10:**
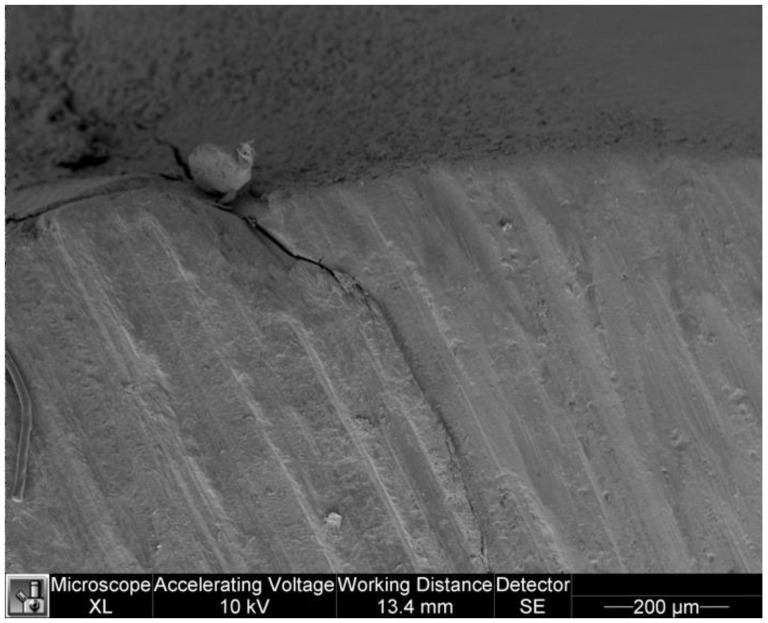
Overhang in a specimen of the control group.

The following tables show the data on the distribution of overhangs between the two groups (Table 5 and Table 6). The *t*-test showed significant differences between SG and CG for the total overhangs (*p* = 0.041). In the SG group, the mean values of the overhangs were larger than in the CG group. Figure 9 and Figure 10 show overhangs in specimens.

### 3.4. Voids

**Figure 11:**
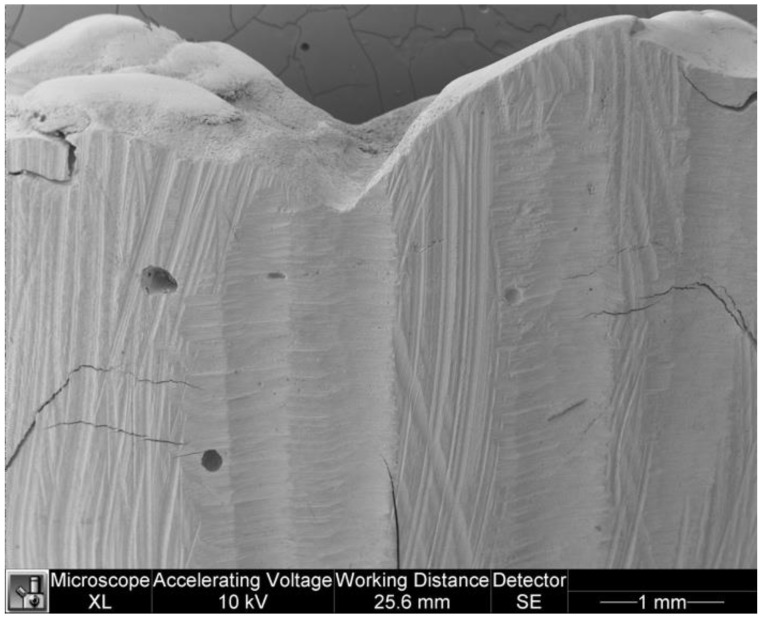
Voids in a specimen of the study group.

**Figure 12:**
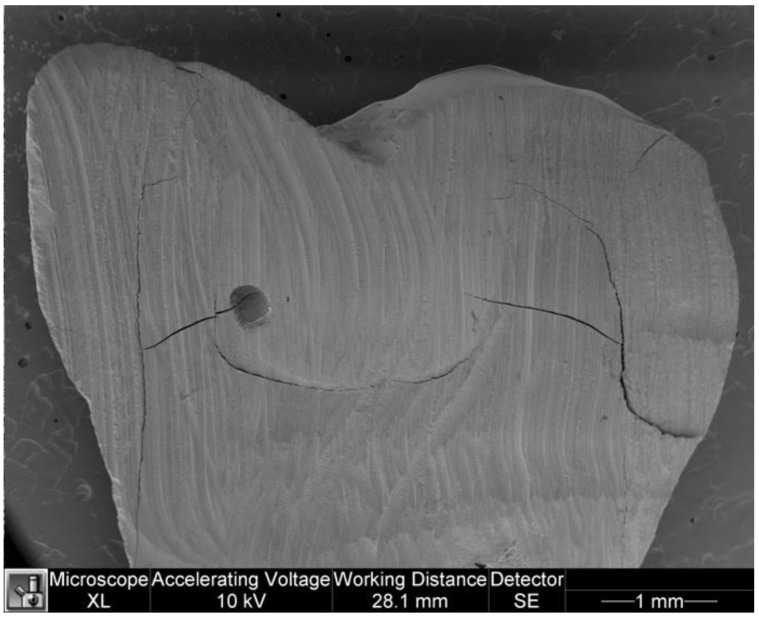
Voids in a specimen of the control group.

The following tables show the data on the presence of voids in the two groups (Table 7 and Table 8). The U Mann-Whitney test showed no statistically significant differences between the two groups (*p* = 0.122). Figure 11 and Figure 12 show voids in specimens.

### 3.5. Operative Times

#### 3.5.1. Restoration Time

The following tables show the Restoration time data for the two groups (Table 9 and Table 10). The *t*-test showed significant differences between SG and CG for this parameter (*p* = 0.021). In the CG group, the mean values for recovery time were lower than in the SG group.

#### 3.5.2. Finishing Time

The following tables show the data on Finishing for the two groups (Table 11 and Table 12). The *t*-test revealed significant differences between SG and CG for this parameter (*p* = 0.011). The mean values highlighted in SG were lower than those of CG.

## 4. Discussion

The aim of the study was to evaluate the advantages and weaknesses of the stamp- technique with regard to the performance of Class I composite restorations in comparison with the traditional restorative technique. Twenty restorations were performed in vitro on extracted teeth and an SEM analysis was conducted for each one. The choice of a small sample like that used in this explorative study was allowed just because the research questions have not previously been studied in depth. An in vitro study was designed to avoid patient-specific factors, i.e., oral hygiene habits or occlusal loading, influencing the results [10]. The measurement of the operative times could also be influenced by patient endurance, so an in vitro study might be a more effective method to avoid bias. Consistency between in vivo and in vitro studies, taking into account the structural phenomena to be observed, ensures a result that corresponds to real conditions observable in clinical practice [12]. Due to the lack of evidence and protocols, the authors decided not to sterilise the teeth before restoration, as this could alter the tooth structures, especially the collagen fibres, which are crucial for the success of the adhesive procedures [14]. A comparison of in vivo and in vitro data revealed that the most valid method for analysing the stability of adhesion over time is sample ageing, a process that makes the results of microleakage and marginal adaptation more clinically relevant [15]. Thermocycling is a commonly used ageing technique in which specimens are subjected to constant and cyclic temperature changes, which affects the tooth-restoration interface in two ways: (1) by accelerating chemical degradation, as hot water accelerates hydrolysis of the interface components, leading to water infiltration and degradation of the adhesive structures; (2) by repeating contraction/expansion processes, as the different thermal coefficients of the composite and the tooth tissue create stresses that lead to cracks and consequently gaps at the interface [16]. The standard ISO TR 11450 (2015) states that 500 cycles of 30″ in water with temperature variations between 5 ± 2 °C and 55 ± 2 °C is an effective ageing procedure useful for simulating long-term microscopic changes and comparing them between different groups [10]. Authors considered thermocycling protocols proposed in the literature are various and greatly different from each other, nevertheless that used in this study was supported by literature and filled the purpose of the research [13]. An extended time of aging was not required to estimate the patterns of a new kind of restoration technique, that is why the thermocycling protocol was limited to 1000 cycles.

Microleakage is a very common blind spot in dental restorations. If the margins of the restoration are not completely sealed, fluids, bacteria and debris can easily penetrate the interface, resulting in secondary cavities, pulpal stimulation with postoperative sensitivity and marginal discolouration.

From the results of the tests performed on the two groups (study and control), there was no statistically significant difference in microleakage of the restorations (*p* = 0.288). We can therefore assume that the stamping technique does not have a critical effect on this aspect. It is plausible to assume that adequate pressure against the still deformable composite in the last 2 mm of the cavity is sufficient to achieve a good seal at the tooth-restoration interface.

Various methods for evaluating microleakage in composite restorations are offered in the literature. In the present study, SEM analysis without dyes was chosen and 40× and 250× images were used to visually evaluate the parameters. One of the most commonly used techniques for the analysis of microleakage is the observation of the penetration of dyes under the stereomicroscope and the light microscope at 40× magnification, a simple technique [17]; however, the authors chose to use SEM as it allows a more accurate measurement even at higher magnifications to better understand the behaviour of composite in very small areas of restorations.

One parameter that could influence the results of the study and needs to be taken into account is the method by which the teeth were cut. In this study, a thin conical diamond drill was used under abundant irrigation. Nevertheless, the high speed of the handpiece could have resulted in high stress levels that may act at the tooth-restoration interface. An alternative method for preparing teeth could be the use of microtomes or cutters with diamond discs, as suggested by some authors [14]. Excellent marginal adaptation depends mainly on the properties of the resins and the adhesive system. Weakness in this area could lead to bacterial infiltration and secondary cavities with resultant damage to the dental pulp [14].

This study did not highlight any significant differences between the two groups studied (*p* = 0.821), however some clarifications are needed.

First and foremost, the mean values of the Marginal Gap variable are higher in the SG, although the statistical differences between the two groups are not significant and this could be due to the small sample. It is to be expected that the accuracy of fit is lower in restorations made with the stamp technique, as the pressure of the occlusal punch on the not yet cured layer of composite (1.5–2 mm) may cause critical points such as excess resin or insufficient fit in the prepared cavity. This can occur especially in anatomically unfavourable conditions (deep and dense pits and fissures). In this particular case, if the basal layer of the cured composite is elevated, the occlusal stamp might not fit accurately, resulting in gaps and poor marginal adaptation (Figure 3, Figure 4 and Figure 5).

However, when analysing the Finishing time data, it is possible to find lower values in the SG. This could be due to insufficient completion with resulting overhanging margins in the study group.

The clinical relevance of overhanging margins depends on the shape and anatomy of the restoration (smooth, rough and voluminous). Rough and voluminous margins or rough overhangs lead to bacterial plaque accumulation and periodontal inflammation. In this regard, reworking, replacement or repair of the restoration might be advisable.

The test performed on the two groups showed statistically significant differences between them (*p* = 0.041) in terms of overhangs. The pressure of the occlusal stamp against the not yet light-cured composite allows excess composite to flow out. In this study, the excess composite was removed after the occlusal punch and teflon were removed before light curing [4,18], however, the literature reports that the excess can be removed with rotary instruments after light curing has been performed while retaining the occlusal punch and Teflon [1,9]. Therefore, further evaluation of the overhangs could be carried out on the samples filled with this method. The current literature does not provide sufficient content for the evaluation of overhangs, the results are mainly focused on amalgam restorations and are not representative for composite fillings [19]; due to this, it was impossible to find a unique method of evaluating the parameter. As mentioned earlier, a lack of filling on the surface or in the thickness of the restoration could compromise its properties, promoting marginal discoloration [20], reducing bond strength, and leading to restoration failure [21]. Our results showed no statistically significant differences between the two groups in terms of the diameter of the voids within the coronal 2 mm of the restoration (*p* = 0.122). This result is really unexpected, since the authors assumed a greater presence of voids larger than 2 mm in the SG because it was suspected that the apposition of the last resin layer with the occlusal punch would entail a greater risk of air bubble incorporation. In particular, it was speculated that using the occlusal stamp pressed against the moldable composite would make it difficult to displace all air bubbles in the last 2 mm. On the other hand, it could be taken into account that the use of a multilayer traditional technique also entails the risk of voids, especially if the resin is not sufficiently pressed. Indeed, voids are mainly caused by the entrapment of air droplets during the filling of the cavity [22], which is why the risk of voids increases with the number of layers. The measuring of bubble size in two dimension is a limitation of this study. Further in-depth analysis to measure the volume of voids may be of interest. In this context, a valuable method could be μ-CT (micro-computed tomography), which allows accurate measurement of void volume without the need to dissect the teeth [21]. Our results are limited to measuring the diameter of voids, which does not allow for accurate assessment of air bubbles, which may have an irregular, not necessarily spherical, morphology. However, a limitation for the μ-CT is that materials that are not radiopaque enough, such as dental adhesives, are difficult to detect in software reconstruction, so determining the differences between adhesives and voids is often difficult. In contrast, SEM analysis at higher magnifications allows deeper evaluation of areas where voids are identified [23]. A combined analysis could represent the gold standard in the investigation of gaps in the thickness of restorations.

Despite the obvious difference of operative times required for in vitro and in vivo restoration, it can be assumed that the present results could be a valuable indicator for the evaluation of times for the two procedures studied here.

Statistical differences between the two groups were found in terms of Restoration time (*p* = 0.021), which was higher in the SG. This is an unexpected datum, considering that in several case reports in the literature, a reduction in time for clinical procedures [1,7,19]. However, most studies in the literature compare operative times for the entire restoration. In this work, the authors made a conscious decision to measure the Restoration time and the Finishing time separately and also to include the time for fabrication of the occlusal stamp, as this affects the duration of the entire procedure.

The results showed a statistical difference between the two groups in terms of Finishing time (*p* = 0.011), with lower mean values in the SG group, probably due to the possibility of avoiding occlusal adjustments made possible by the stamp technique [7]. The real advantage lies in the possibility of achieving a good ratio between cusp and pit [18]. Considering all this, further studies are needed to accurately determine the possibilities and advantages of the stamp technique, since it is a newly introduced process, and the literature does not yet contain sound instrumental studies and follow-ups.

## 5. Conclusions

Within the limitations of this exploratory study, the stamp technique does not appear to have any critical aspects in terms of restoration durability, such as microleakage, marginal adaptation and filling defects. However, this technique promotes the formation of large overflowing margins that require a careful finishing phase. Further studies with a larger sample are needed to improve these findings.

## Figures and Tables

**Table 1:** Microleakage in the study group.

Sample	Left Microleakage (mm)	Right Microleakage (mm)	Microleakage (mm)
L9T	0.69	3.02	3.02
L10T	0.46	0	0.46
L11T	0.37	0	0.37
L12T	0	0	0
L13T	0.1	0.02	0.1
L14T	0.33	0	0.33
L21T	0	0	0
L22T	0.01	0.39	0.39
L23T	0	0	0
L24T	0	0	0
**Mean**	0.20	0.34	**0.47**
**Median**	0.06	0.00	**0.22**
**Standard Deviation**	0.19	0.13	**0.92**

**Table 2:** Microleakage in the control group.

Sample	Left Microleakage (mm)	Right Microleakage (mm)	Microleakage (mm)
L15C	0	1.93	1.93
L16C	0.15	0	0.15
L17C	1.82	0	1.82
L18C	0.32	0	0.32
L19C	0.06	0	0.06
L20C	0.12	0	0.12
L25C	0.3	0.17	0.3
L26C	2.12	0.24	2.12
L27C	0.16	0	0.16
L28C	0	0.44	0.44
**Mean**	0.51	0.28	**0.74**
**Median**	0.16	0.00	**0.31**
**Standard Deviation**	0.81	0.16	**0.85**

**Table 3:** Marginal adaptation in the study group.

Sample	Left Marginal Gap (µm)	Right Marginal Gap (µm)	Marginal Gap (µm)
L9T	6.68	20.59	20.59
L10T	5.8	9.14	9.14
L11T	56	5.16	56
L12T	0	2.6	2.6
L13T	4.14	6.9	6.9
L14T	11.93	6.98	11.93
L21T	3.92	2.6	3.92
L22T	44.97	33.14	44.97
L23T	3.35	16.08	16.08
L24T	9.38	50.89	50.89
**Mean**	14.62	15.41	**22.30**
**Median**	6.24	8.06	**14.01**
**Standard Deviation**	**19.36**	**15.69**	**20.42**

**Table 4:** Marginal adaptation in the control group.

Sample	Left Marginal Gap (µm)	Right Marginal Gap (µm)	Marginal Gap (µm)
L15C	2.23	13.87	13.87
L16C	12.07	5.52	12.07
L17C	31.21	3.98	31.21
L18C	13.84	2.37	13.84
L19C	2.12	2.09	2.12
L20C	5.11	1.39	5.11
L25C	12.48	8.67	12.48
L26C	15.09	7.94	15.09
L27C	16.19	0	16.19
L28C	8.48	28.44	28.44
**Mean**	11.88	7.43	**15.04**
**Median**	12.28	4.75	**13.86**
**Standard Deviation**	8.51	8.47	**8.98**

**Table 5:** Overhangs in the study group.

Sample	Right Overhangs (µm)	Left Overhangs (µm)	Marginal Overhangs (µm)
L9T	19.03	97.48	97.48
L10T	30.73	28.84	30.73
L11T	29.72	117.26	117.26
L12T	0	44.25	44.25
L13T	52.01	69.03	69.03
L14T	65.33	24.11	65.33
L21T	5.19	28.37	28.37
L22T	50.3	116.57	116.57
L23T	85.49	18.91	85.49
L24T	25.45	68.64	68.64
**Mean**	36.33	57.33	**72.32**
**Median**	30.23	44.25	**68.84**
**Standard Deviation**	26.83	38.35	**32.16**

**Table 6:** Overhangs in the control group.

Sample	Right Overhangs (µm)	Left Overhangs (µm)	Marginal Overhangs (µm)
L15C	5.95	31.69	31.69
L16C	23.03	43.77	43.77
L17C	44.44	24.26	44.44
L18C	84.5	3.35	84.5
L19C	0	16.9	16.9
L20C	6.62	0	6.62
L25C	35.28	25.3	35.28
L26C	42.25	73.38	73.38
L27C	52.87	0	52.87
L28C	10.4	55.7	55.7
**Mean**	30.53	27.44	**44.52**
**Median**	29.16	24.78	**44.11**
**Standard Deviation**	26.53	24.51	**23.75**

**Table 7:** Voids detected in the study group.

Sample	Presence of Voids	Presence of Voids in the Last Coronal 2 mm	Largest Diameter of Voids (mm)	Diameter of Voids in the Last Coronal 2 mm
L9T	Yes	Yes	0.17	0.17
L10T	Yes	Yes	0.37	0.37
L11T	Yes	Yes	0.29	0.29
L12T	Yes	No	0.25	0
L13T	Yes	Yes	0.29	0.29
L14T	Yes	No	0.06	0
L21T	Yes	Yes	0.15	0.15
L22T	Yes	Yes	0.15	0.15
L23T	Yes	Yes	0.19	0.19
L24T	Yes	Yes	0.13	0.13
**Mean**			**0.21**	**0.17**
**Median**			**0.18**	**0.16**
**Standard Deviation**			**0.09**	**0.12**

**Table 8:** Voids detected in the control group.

Sample	Presence of Voids	Presence of Voids in the Last Coronal 2 mm	Largest Diameter of Voids (mm)	Diameter of Voids in the Last Coronal 2 mm
L15C	Yes	No	0.03	0
L16C	Yes	Yes	0.1	0.1
L17C	Yes	Yes	0.16	0.16
L18C	Yes	Yes	0.11	0.11
L19C	Yes	No	0.24	0
L20C	Yes	No	0.05	0
L25C	Yes	Yes	0.18	0.18
L26C	Yes	Yes	0.38	0.38
L27C	Yes	No	0.37	0
L28C	Yes	No	0.03	0
**Mean**			**0.17**	**0.09**
**Median**			**0.14**	**0.05**
**Standard Deviation**			**0.13**	**0.12**

**Table 9:** Restoration time data in the study group.

Sample	Stamp Preparation (s)	Restorative Procedures (s)	Restoration Time (s)
L9T	803	1035	1838
L10T	511	549	1061
L11T	806	1190	1996
L12T	610	930	1540
L13T	625	530	1155
L14T	639	948	1587
L21T	580	815	1395
L22T	482	522	1004
L23T	479	755	1234
L24T	395	635	1030
**Mean**	**593**	**790.9**	**1384**
**Median**	**595**	**785**	**1314.5**
**Standard Deviation**	**135.11**	**232.84**	**348.86**

**Table 10:** Restoration time data in the control group.

Sample	Restoration Time (s)
L15C	851
L16C	734
L17C	831
L18C	1395
L19C	1081
L20C	998
L25C	1490
L26C	668
L27C	1290
L28C	912
**Mean**	**1025**
**Median**	**955**
**Standard Deviation**	**282.85**

**Table 11:** Finishing time data in the study group.

Sample	Finishing Time (s)	Polishing Time (s)	Finishing + Polishing Time (s)
L9T	63	59	122
L10T	22	49	71
L11T	40	44	84
L12T	12	12	24
L13T	10	15	25
L14T	15	12	27
L21T	14	15	29
L22T	20	30	50
L23T	17	15	32
L24T	42	38	80
**Mean**	25.5	28.9	**54.4**
**Median**	18.5	22.5	**41.00**
**Standard Deviation**	17.21	17.55	**33.47**

**Table 12:** Finishing time data in the control group.

Sample	Finishing Time (s)	Polishing Time (s)	Finishing + Polishing Time (s)
L15C	63	59	122
L16C	46	34	80
L17C	72	59	131
L18C	45	35	80
L19C	33	26	59
L20C	31	58	89
L25C	40	39	79
L26C	32	37	69
L27C	58	32	90
L28C	50	55	105
**Mean**	47	28.9	**90.4**
**Median**	45.5	22.5	**84.5**
**Standard Deviation**	13.91	17.55	**22.73**

## Data Availability

Not applicable.
